# 

**Published:** 1917-09

**Authors:** 


### ANNOUNCEMENTS

MICHIGAN STATE BOARD OF DENTAL EXAMINERS. The next session of the Michigan State Board of Dental Examiners for the examination of candidates wishing to practice dentistry in the state of Michigan, will be held in the city of Ann Arbor (Dental College Building), the week of November 5 to 10 inclusive.

Applications should be made early as all preliminaries must be complete and in the hands of the secretary not less than ten (10) days prior to the examination.

Photographs and itemized preliminary educational qualifications required of all candidates.

For all further information address, E. O. Gillespie, Secretary and Treasurer, Stephenson, Mich.

---

### TWENTY-FIRST ANNUAL SESSION OF THE NATIONAL DENTAL ASSOCIATIONS

New York City, Oct. 22-26, 1917

(All sessions of the Board of Trustees, House of Delegates, General Sessions, Section Meetings, Clinics (except Surgical Clinics), exhibits will be held in Hotel Astor.)

The following additions may be announced to the program as already published. Monday, Oct. 22. Registration. (Laurel Room, First Floor.) First Session, Board of Trustees, 11 a. m. (Rose Room—First Floor.) House of Delegates—Opening Session, 2 p. m. All Sessions of the House of Delegates will be held in the Rose Room, First Floor; Second Session, Board of Trustees, 4 p. m. (Rose Room—First Floor.)

*First General Session*—Hotel Astor. (Grand Ball Room—First Floor.) Tuesday, Oct. 23, 9 a. m. Invocation; Address of Welcome, Gov. Charles S. Whitman, on behalf of State, Albany, N. Y.; Mayor John P. Mitchel, on behalf of City, New York City; President's Address, LaFayette L. Barber, Toledo, O.; Oration, E. C. Rosenow

(M.D.), Rochester, Minn., a research member of the Memorial Institute for Infectious Diseases, Chicago, and Professor of Experimental Bacteriology in the University of Minnesota and the Mayo Institute.

*Second General Session*—Tuesday, Oct. 23, 8 p. m. (Grand Ball Room—First Floor.) Research Department. Pathological, Bacteriological and Clinical Studies. "Dental Caries," by Russell W. Bunting and U. G. Rickert (B.S., M.A.). Work done in, and with the assistance of, the University of Michigan. "A Study of the Pathology of the Peridental Membrane," by Frederick B. Noyes. Work done in, and with the assistance of, the University of Illinois. "The Histopathology of Chronic Periodontitis and the Pathogenesis of Dental Root Cysts," by Thomas B. Hartzell and Arthur T. Henrici (M.D.). Work done in, and with the assistance of the University of Minnesota. "A Comparative Study of Oral Focal Infections," by Weston A. Price and Milton J. Damlos (D.D.S.). Research Institute Laboratories, Cleveland, Ohio.

*Third General Session*—Wednesday, Oct. 24, 8 p. m. (Grand Ball Room—First Floor.) Herbert L. Wheeler, Chairman, New York City. Dental Surgery and Restorative Prostheses in the European War. Symposium, "Dental Surgery and Restorative Prostheses in the European War." "War Dental Surgery," by ———; "War Dental Restorative Prostheses," by ————. It is expected that Mr. Robert Bacon, Ex-Ambassador to France, will preside, and that the Surgeons General of both the Army and Navy will attend. A description of the work done in Europe will be given by one who has had experience there. It is anticipated that both the Medical and Dental Professions will be represented. There will also be pictures of "War Surgery." Herbert L. Wheeler, Chairman.

*Fourth General Session*—Thursday, Oct. 25, 8 p. m. (Grand Ball Room—First Floor.) Oral and Dental Hygiene—Mass Meeting. Charles H. Oakman, Chairman, Detroit, Mich. "The Importance of Dental Infections as Related to Urinary Tract Diseases, with Particular Reference to Ureter Stricture and Its Sequelæ." Guy L. Hunner (S.B., M.D.), Johns Hopkins University, Baltimore, Md.

*Research Department*—Wednesday, Oct. 24, 1:30 p. m. "Root Canal Fillings," by John R. Callahan. Work done in, and with the assistance of, the Cincinnati General Hospital. "Studies Upon the Bacteriology of Dental Caries," by Percy R. Howe. Work done in, and with the assistance of, the Forsyth Dental Infirmary. "Quantitative Determinations of Certain Organic Substances in Saliva and Their Relation to Oral Conditions," by John A. Marshall (M.S., D.D.S.). Work done in, and with the assistance of, the University of California. "Electrolytic Medication—Physiological and Dental Aspects," by Samuel E. Pond (B.H., A.M.), and Weston A. Price, Research Institute Laboratories, Cleveland, Ohio. Thursday, Oct. 25, 9 a. m. Chemical and Metallurgical Studies. "Dental Cements," by Marcus L. Ward. Work done in, and with the assistance of, the University of Michigan. "Progress of the Investigation of Mottled Enamel," by Frederick S. McKay, Colorado Springs, Colo. "Studies of Internal Secretions in Their Relation to the Development and Condition of the Teeth," by William J. Gies (M.D., Ph.D.). Work done in, and with the assistance of, the Columbia University. "The Relative Efficiency of Medicaments for the Sterilizing of Tooth Structures," by Matilda Moldenhauer (A.B., M.S.) and Weston A. Price, Research Institute Laboratories, Cleveland, Ohio.

*Preparedness League of American Dentists*—Tuesday, Oct. 23, 1:30 p. m. (College Hall—Eighth Floor.) "The Relations of the Dental to the Medical Corps of the Army," by Victor C. Vaughan (M.D.), Ex-President of the American Medical Association, Member General Medical Board of the Council of National Defense, Washington, D. C. "The Value of the Educational Work as Being Executed by the Preparedness League of American Dentists," by Frank M. Casto, Cleveland, Ohio. "Report of Sectional Units of League by States—A to N Inclusive." Thursday, Oct. 25, 1:30 p. m. "Stereopticon Lecture on Fractures of the Bones of the Face." (Synopsis: This lecture will include the consideration and treatment of gun shot wounds; removing foreign substances; the sterilization of wounds; the proper adjustment of fragments and the means employed to mobilize them. In the treatment of injuries of the face, the primary step should be after removing foreign substances, to establish as nearly as possible, asepsis. The use of grafts of bone, modeling compound, hard rubber and metals to replace lost parts.) "The Dental Ambulance in War," by George B. Hayes, Neuiely, (Paris) France. "Report of Sectional Units by States—M to Z Inclusive." (An interesting Preparedness Exhibit will be shown, including Dental Ambulances, Base Hospital, Field Hospital and First Aid Equipments; in fact, everything relative to the service of the Officers' Reserve Corps, Dental Section and Red Cross Officers will be present to give instruction and information. The ambulances shown will be sent to France as the gift of members of the Preparedness League and appreciative patients.)

*Anesthetists Section*—Wednesday, Oct. 24, 1:30 p. m. (East Ball Room—Eighth Floor.) "The Teaching of Local Anesthesia," by Theo. Blum, New York City. "The Relative Toxicity of Local Anesthetics," by George B. Roth, Washington, D. C. "Cause of Failure and Untoward Sequelæ in Conductive Anesthesia," by Richard H. Riethmiller, Montclair, N. J. (Illustrated by Moving Picture Film.) Thursday, Oct. 25, 9 a. m. "After-pain in Local and General Anesthesia," by A. E. Hertzler, Kansas City, Mo. "General Anesthesia for Oral Surgery," by Herbert A. Potts, Chicago, Ill.

*State Society Officers Section*—Wednesday, Oct. 24. 9 a. m. (College Hall—Eighth Floor.) "Post Graduate Course," by B. L. Shobe, Tulsa, Okla. "State Legislation from a Dental Standpoint," by A. H. Reynolds, Philadelphia, Pa. "Taking an Invoice of Our State Societies,'' by Otto U. King, Huntington, Ind. Wednesday, Oct. 24, 1:30 p. m. ''Organization,'' by J. P. Luthringer, Peoria, Ill. Symposium—''Method Used in Handling the Mid-Winter Dental Clinic, Atlanta Dental Society,'' by Thomas P. Hinman, Atlanta, Ga.; S. M. Cameron, Philadelphia, Pa.; Benjamin Sandy, Minneapolis, Minn.; Henry L. Whipple, Quincy, Ill. (These papers will be limited to ten minutes each, and followed by general discussion.) R. Ottolengui, Publicity Committee.

